# The New York State Convention

**Published:** 1884-01

**Authors:** 


THE NEW YORK STATE CONVENTION.—In response to a call issued by Mr. T. E. Daniels, of Chicago, a number of veterinarians from various parts of the State, chiefly from N. Y. city and Brooklyn, assembled at the New York Academy of Medicine, October 24th, and proceeded to the organization of a State Convention or Association.

The following gentlemen were elected permanent officers of the organization:

*President*—James Cosgrove, D.V.S., of N. Y.

*First Vice-President*—E. M. Fitzgerald, D.V.S., of Greenpoint, N. Y.

*Second Vice-President*—Thos. Robertson, M.R.C.V.S., of Brooklyn.

*Third Vice-President*—W. E. A. Cuff, D.V.S., of New York.

*Recording Secretary*—E. S. Breeder, D.V.S., of New York.

*Corresponding Secretary*—E. W. Douglass, D.V.S., of Brooklyn.

*Treasurer*—John Cuff, D.V.S., of New York.

The Society adjourned at the call of the President.

